# The increasing potential of nuclear medicine imaging for the evaluation and reduction of normal tissue toxicity from radiation treatments

**DOI:** 10.1007/s00259-021-05284-5

**Published:** 2021-03-09

**Authors:** V. Mohan, N. M. Bruin, J. B. van de Kamer, J.-J. Sonke, Wouter V. Vogel

**Affiliations:** 1 Department of Nuclear Medicine, The Netherlands Cancer Institute, Plesmanlaan 121, 1066 CX Amsterdam, The Netherlands; 2 Department of Radiation Oncology, The Netherlands Cancer Institute, Amsterdam, The Netherlands

**Keywords:** Toxicity, Molecular imaging, Radiotherapy, Radionuclide therapy

## Abstract

Radiation therapy is an effective treatment modality for a variety of cancers. Despite several advances in delivery techniques, its main drawback remains the deposition of dose in normal tissues which can result in toxicity. Common practices of evaluating toxicity, using questionnaires and grading systems, provide little underlying information beyond subjective scores, and this can limit further optimization of treatment strategies. Nuclear medicine imaging techniques can be utilised to directly measure regional baseline function and function loss from internal/external radiation therapy within normal tissues in an in vivo setting with high spatial resolution. This can be correlated with dose delivered by radiotherapy techniques to establish objective dose-effect relationships, and can also be used in the treatment planning step to spare normal tissues more efficiently. Toxicity in radionuclide therapy typically occurs due to undesired off-target uptake in normal tissues. Molecular imaging using diagnostic analogues of therapeutic radionuclides can be used to test various interventional protective strategies that can potentially reduce this normal tissue uptake without compromising tumour uptake. We provide an overview of the existing literature on these applications of nuclear medicine imaging in diverse normal tissue types utilising various tracers, and discuss its future potential.

## Introduction

Radiation as a treatment modality for cancer has existed for over a hundred years [1], and presently in many forms. External beam radiotherapy (EBRT), brachytherapy and radionuclide therapy (RNT) have each proved to be excellent tools in the curative and palliative treatment of a variety of cancers. The core principle behind radiation therapy, i.e. DNA strand damage as a result of direct ionisation by incoming particle radiation, or indirectly via the generation of free radicals by said particles, is however not specific to cancerous tissue [2]. Radiation is indiscriminate, and as such affects normal healthy tissue as well. Modern radiation therapy exploits differences in DNA repair proficiency and strives to maximise the deposition of radiation dose in cancerous tissue while minimising the dose in normal tissue.

For EBRT, the traversal of radiation through normal tissue is inevitable, since most cancers are internal to the human body and the nature of interactions with tissue are stochastic. Advancements in treatment delivery, from conformal radiotherapy to intensity modulated radiotherapy (IMRT) and volumetric arc therapy (VMAT), as well as imaging, which has enabled the advent of image-guided and adaptive radiotherapy, have greatly reduced normal tissue damage from EBRT. Proton and carbon ion therapies have the potential to reduce the dose in traversed normal tissue even further. Toxicities still occur however and are more common in patients that receive concurrent chemotherapy with radiation. Brachytherapy delivers its radiation dose directly within the tumour; however, its applications are site-restricted and local toxicity still occurs due to the pathlength of emitted electrons and photons reaching the surrounding normal tissues. RNT targets and inflicts radiation damage specifically in cancerous tissue by exploiting cellular mechanisms, though non-target normal tissues that present the same mechanisms often fall victim to toxicity, and again local toxicity may still occur in the surrounding normal tissues due to the pathlength of radiation [2].

### Early versus late toxicity

Early toxicity is a result of normal tissue damage that manifests itself within the first few weeks or months of therapy and is usually transient. Late toxicity occurs months or years after treatment, and can be permanent. The type of toxicity depends on the turnover (proliferation and apoptosis) of the tissue type and is organ-specific. In general, early toxicity is characterised by inflammation related to cellular damage and cytokine release in rapidly proliferating tissues, and late effects are characterised by fibrosis and function loss [3]. Early and late toxicities may overlap, and the former may develop into the latter.

An example of early toxicity from head and neck EBRT is mucositis, which can induce complaints of dysphagia and xerostomia. When this acute toxicity resolves after treatment, but the involved tissues are affected by subsequent fibrosis, dysphagia and xerostomia can also become prominent late effects. In parallel, early toxicity from RNT with radioactive iodine (^131^I) for thyroid cancer can include sialadenitis, and late effects due to fibrosis in the salivary glands can also include dysphagia and xerostomia.

### Evaluation of toxicity

Early and late radiation-induced toxicity is usually evaluated through the use of grading systems and questionnaires. The Radiotherapy Oncology Group (RTOG)/European Organisation for Research and Treatment of Cancer (EORTC) scale was the first to see widespread use. The Late Effects Normal Tissues (LENT)-Subjective, Objective, Management, Analytic (SOMA) scale was then later introduced, but was ultimately incorporated into the Common Terminology Criteria for Adverse Events (CTCAE) grading system. The CTCAE is becoming the most widely adopted toxicity grading system [4, 5]. It is comprehensive and covers both early and late effects for all treatment sites. The toxicity grade is selected based on a description of symptoms, anomalies visible on scans or measurements of values from lab tests.

The aforementioned systems are observer/physician rated and as such do not take into account the subjective feeling of the patient. Studies have shown that physicians often underreport the subjective toxicity experienced by patients, and some have shown that patient-reported toxicity correlates more with objective measurements than physician-reported toxicity does [6–8]. Toxicity reporting in recent years has therefore been supplemented with patient-reported outcomes. The patient-reported outcomes (PRO)-CTCAE was introduced in 2016 as a companion to the CTCAE system. The EORTC general and site-specific quality of life questionnaires are often used for patient-reported quality of life. Other questionnaires that report a specific outcome, such as the Groningen Radiotherapy-Induced Xerostomia or Expanded Prostate Index Composite questionnaires, have also been developed and validated by different groups [9, 10].

Patient-reported outcomes, while complimentary and expansive to the perspective of toxicity, are nonetheless still subjective to the individuals reporting them. The use of functional tests, which are sometimes part of toxicity grading, is preferable as they are objective in nature. Examples include blood cell count tests, estimated glomerular filtration rate (GFR), salivary flow rates and pulmonary function tests. Some limitations to these tests are that they are often subject to natural biological variation (reducing repeatability), lack standardisation of instruments and measurement practices (reducing reproducibility), and provide no spatial information. In addition, many of these tests have an invasive nature and may themselves cause discomfort or pain.

### Imaging to evaluate toxicity

Imaging therefore presents itself as a good candidate to measure toxicity objectively, locally, in vivo and non-invasively. The ability to visualise and quantify local effects of radiation dose on the voxel level provides new insight into the dose-effect relationships beyond dose-volume effects. Anatomical imaging with computed tomography (CT) and magnetic resonance imaging (MRI) has been widely used to do so. Shrinkage of parotid gland volume, fibrosis in the lung and thickening of pericardium are just some of the normal tissue toxicity effects that can be visualised [11–13]. Anatomical imaging is, however, insufficient for evaluating many important aspects of toxicity. Instead, a quantifiable measure of decrease in organ function is desirable. Beyond morphological changes, CT or MRI can be used quantitatively as well, for example by looking at changes in Hounsfield units or apparent diffusion coefficient in salivary glands post-therapy as a marker of functional tissue loss [14–16]. 4DCT has been used to measure ventilation changes in the lung [17], but applications to other organs are limited.

Nuclear medicine imaging techniques have an advantage here and show promise, since they by principle are functional modalities, using radioactive pharmaceuticals to map physiological processes. Moreover, changes in function often precede anatomical changes, thereby allowing for evaluation of biological changes in tissues on shorter timescales [18]. This is typically applied for evaluating the response of tumours to treatment. The challenge in evaluating toxicity, however, is in finding or developing tracers that are substantially taken up by healthy or damaged normal tissue. The decay mode of the radionuclide in the tracer used determines the modality of the scan that can be made. Photons emitted from gamma emitters inside a patient, like technetium-99m, are detected by a gamma camera, with which 2D planar scintigraphy or 3D single-photon emission computed tomography (SPECT) images can be made. Positron emitters, like fluorine-18, are imaged in 3D using positron emission tomography (PET), whereby the photon pairs created from the annihilation of positrons inside a patient are coincidentally detected. Each technique has its own advantage when it comes to resolution, quantification, price, availability and convenience. Regardless of the technique employed, changes in the distribution or dynamics of such a radioactive tracer, when compared to a baseline control scan, forms the principle by which tissue toxicity can be evaluated. This can be further used to derive local dose-effect relationships and correlated with clinical outcomes.

For EBRT, this application of molecular imaging can contribute to the development of improved dose prescriptions for normal tissues that may subsequently help to reduce unnecessary toxicity for future patients. In the case of RNT, we can go a step further. Not only can toxicity from a cycle of RNT be evaluated by making a diagnostic scan before and after therapy, which may then also be used to plan a successive cycle, but imaging can potentially also assist in the development of new strategies to influence tracer biodistribution and thereby reduce toxicity. Unlike in EBRT where dose delivered can be controlled carefully, in RNT, control of the biodistribution of the tracer (and therefore the dose delivered to tissues) is limited. However, most therapeutic radiopharmaceuticals have diagnostic analogues that are used to assess a patient’s tumour load. These diagnostic counterparts are also taken up by the same normal tissues as the therapeutic ones, assuming that the uptake and dynamics of the two radiopharmaceuticals are similar. With this type of diagnostic biodistribution imaging, strategies to protect normal tissues from unwanted uptake and dose can be tested without the need of a therapeutic dose.

## Overview of possibilities

The goal of this section is to provide a comprehensive overview and brief discussion of past and recently published applications of nuclear medicine imaging techniques, namely planar scintigraphy, SPECT and PET, for the evaluation and reduction of toxicity from EBRT and RNT (brachytherapy is not discussed) in a variety of normal tissues. Some reviews in the past have reported on this but were either focused on a single tracer, a single imaging or treatment modality, or a single evaluated tissue type. Given the vast number of cancer types and treatment sites, as well as the organs at risk associated with them, this list is by no means exhaustive, but merely exemplary in nature. In this section, we divide the discussion based on tissue type, and proceed in alphabetical order. An overview of some of the discussed toxicities and their incidence rates is reported in Table 1.

**Table 1:** Incidence rates of some toxicities that are discussed

Tissue type	Primary cancer type	Treatment type	Toxicity type	Incidence	Study
Brain	Lung, breast, etc.	WBRT and/or SRS	≥ Grade 3 neurological	2%	Andrews et al. [19]
Bone marrow	Cervical	IMRT	≥ Grade 3 haematological	27%	Rose et al. [20]
	Neuroendocrine	^177^Lu/^90^Y-DOTA-TATE/TOC RNT		10%	Bodei et al. [21]
	Prostate	^177^Lu-PSMA RNT		12%	Rahbar et al. [22]
Heart	Lung	3D conformal RT	≥ Grade 3 cardiac	11%	Dess et al. [23]
Kidneys	Neuroendocrine	^177^Lu/^90^Y-DOTA-TATE/TOC RNT	≥ Grade 1 renal	35%	Bodei et al. [21]
	Prostate	^177^Lu-PSMA RNT	≤ Grade 2 renal	12%	Rahbar et al. [22]
Liver	Liver	SBRT	≥ Grade 3 hepatic	7%	Bujold et al. [24]
		^90^Y SIRT		21%	Strigari et al. [25]
Lungs	Lung	IMRT	≥ Grade 3 radiation pneumonitis	4%	Chun et al. [26]
		SBRT		2%	Chaudhuri et al. [27]
Salivary glands	Head and neck	IMRT	≥ Grade 2 xerostomia	15–39%	Marta et al. [28]
	Prostate	^177^Lu-PSMA RNT	≤ Grade 2 xerostomia	8%	Rahbar et al. [22]
		^225^Ac-PSMA RNT	≥ Grade 3 xerostomia	50%	Kratochwil et al. [29]
	Thyroid	^131^I RNT	≥ Grade 1 xerostomia	16–54%	Clement et al. [30]

### Bone marrow

**Fig. 1:**
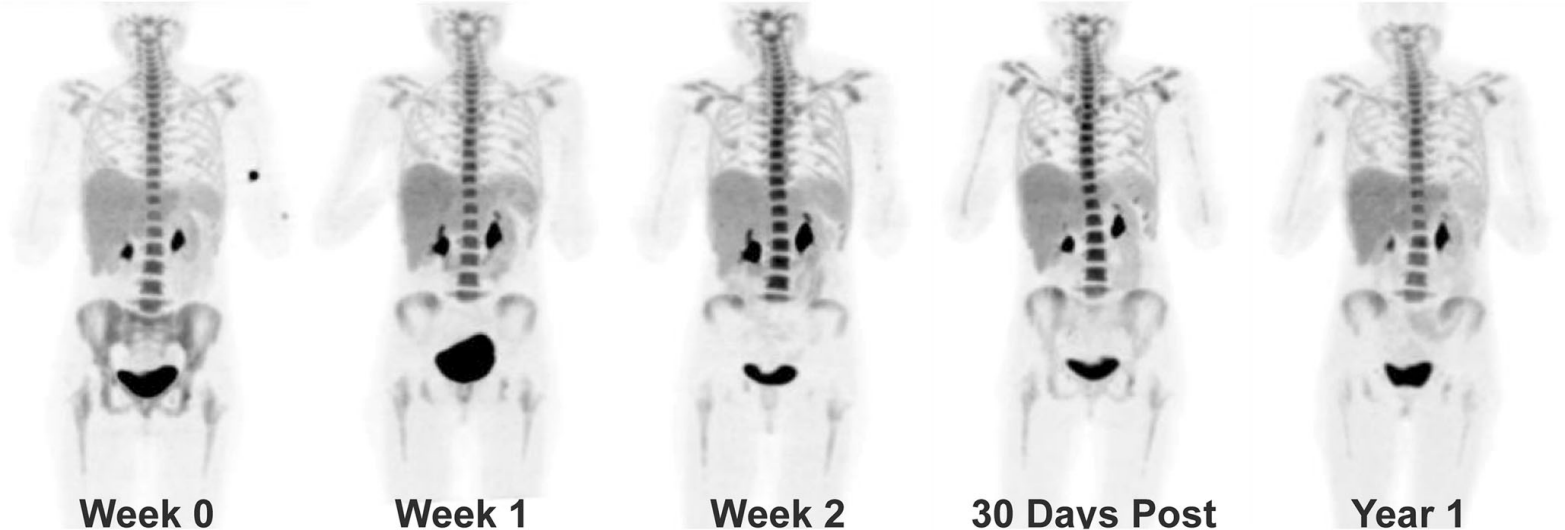
Longitudinal whole-body [^18^F]FLT-PET images acquired for a patient who received RT for pelvic cancer. This image was reproduced from McGuire et al. with permission [35]

Bone marrow tissue is extremely sensitive to radiation, and is distributed throughout the body, mainly in the interior of flat bones and the ends of long bones. Haematological toxicity from EBRT may be minor if the volume of bone marrow irradiated in the target region is limited, and the other locations are spared. This toxicity is often quantified with blood tests; however, its distribution and local extent can only be visualised and measured using functional imaging techniques like 3′-deoxy-3′-[^18^F]fluorothymidine (FLT) and [^18^F]fluoro-2-deoxyglucose (FDG) PET [31–34]. While [^18^F]FDG PET is a more routinely carried out scan and provides information about the metabolic activity of marrow content, it is non-specific. [^18^F]FLT-PET, a modality that reflects cell proliferation and DNA synthesis, is far better in identifying vital bone marrow regions. One study that incorporated [^18^F]FLT-PET scans to spare bone marrow in pelvic cancer patients receiving IMRT (Fig. 1) found that reductions in uptake correlated significantly with toxicity outcomes from blood tests after treatment. While a significant reduction in dose to bone marrow regions was achievable, the sensitivity of the tissue to radiation (near 50% reduction in uptake within the first 2 weeks of therapy after receiving a mere 4Gy) limited its effect on the reduction of toxicity [35].

Bone marrow toxicity also occurs in virtually all forms of RNT due to the circulation of radiopharmaceuticals in the body. Therefore, in contrast to EBRT, all locations of bone marrow in the body are, in principle, irradiated. [^177^Lu]Lu-octreotate (DOTA-TATE) therapy, used for treating neuroendocrine tumours, is one such RNT, where the bone marrow, along with the kidneys, is often considered a dose-limiting organ. Owing to the theragnostic nature of the ^177^Lu-DOTA-TATE molecule, SPECT images can also be made alongside its therapeutic action to verify in vivo dosimetry and biodistribution. Two studies used this principle and found the estimated dose to the bone marrow to be much higher than blood-based dosimetry methods [36, 37]. One of them also showed correlations between bone marrow dose, and reduction in platelet counts [37]. RNTs are usually delivered over multiple cycles, and these post-therapy scans can be used to plan subsequent therapy cycles and even assess potential toxicity between them. Recently, this toxicity was also visualised in mice treated with ^177^Lu-DOTA-TATE, using [^18^F]FLT-PET, where a reduction in 50% of SUV_max_ was seen [38]. There is an increasing use of RNT for patients with extensive bone metastases from castration-resistant prostate cancer, for example with ^223^Ra or radiolabelled prostate-specific membrane antigen (PSMA) ligands (possibly even subsequently) [39, 40]. These tracers do not actively target haematological cells, but the circulation phase and accumulation in normal bone or bone metastases can result in significant dose to bone marrow [41]. A study used baseline [^18^F]fluorocholine(FCH) PET, typically used to detect metastatic recurrent prostate cancer, to determine bone tumour infiltration and was thereby able to predict haematological toxicity in metastatic prostate cancer patients who received ^223^Ra therapy [42]. Imaging the normal tissue directly with [^18^F]FLT-PET could possibly be used to evaluate toxicity in these cases as well. There thus seems to be potential value in incorporating molecular imaging in the development of improved or personalised RNT strategies.

### Brain

Radiation damage to the brain can result in neurocognitive and motor deficits, oedema and radiation necrosis. In addition to regular RT techniques, whole brain radiation therapy (WBRT) and stereotactic radiosurgery (SRS) are also commonly used to treat primary and metastatic brain tumours. Since the brain depends on glucose for its metabolism, this makes [^18^F]FDG a strong choice for investigating the local effects of radiation. One study found a reduction in the uptake of [^18^F]FDG in normal brain tissue (2–6% reduction in regions receiving more than 40 Gy), measured at 3 weeks and 6 months after treatment, in patients treated with EBRT for a primary brain tumour, that correlated with symptoms of neurocognitive dysfunction. The same study also noted an initial increase in cerebral blood flow in the same regions (< 10%), which later subsided at 6 months post-treatment, measured with [^15^O]H_2_O PET [43]. This mirrors results found in an earlier study that measured cerebral blood flow during and 3 months after radiotherapy using ^133^Xe SPECT [44]. Another study that looked at changes in [^18^F]FDG uptake in the brain before and after prophylactic WBRT for small cell lung cancer found asymmetric unilateral changes, while a bilateral decrease was expected. They claimed this suggests functional changes rather than normal cellular toxicity [45]. In another study, adult survivors of acute lymphoblastic leukaemia treated with WBRT were found to have increased metabolic activity in many parts of the brain, contradictory to the findings of the previous studies, and this strongly associated with neurocognitive impairment from evaluations [46]. Although these findings are interesting, implications for treatment planning or optimization for quality of life have not yet been studied. Tumour recurrence and radiation necrosis after RT can often be difficult to distinguish on conventionally used contrast-enhanced MRI scans. PET with O-(2-[^18^F]fluoroethyl)-L-tyrosine (FET), an amino acid tracer, can contribute to solving this problem. Although unable to evaluate toxicity by itself, in conjugation with MRI, it can verify the presence of necrosis by eliminating the possibility of a recurrence [47].

### Heart

Cardiotoxicity from radiation can be measured in many ways. Studies that made use of [^18^F]FDG PET found increased uptake in cardiac wall regions after thoracic radiotherapy, especially in volumes that received more than 20 Gy [48, 49]. Myocardial perfusion imaging, initially with ^201^Tl, a physiological potassium analogue, and later with superior technetium-based tracers (like [^99m^Tc]Tc-sestamibi or [^99m^Tc]Tc-tetrofosmin) have also been used to measure radiation-induced cardiotoxicity in breast cancer patients [50], finding higher abnormality rates in left-sided breast cancer patients (71% of left-sided breast cancer patients and 17% of right-sided breast cancer patients) [51]. More recently, there are studies that have used PET perfusion tracers like [^15^O]H_2_O and [^15^N]NH_3_ to do the same [52–54]. One study [52] found statistically significant changes in perfusion occurred in 86% of patients 2 months after RT, and were independent of left- or right-sided breast cancer. Though the magnitude of these changes was not predictive for toxicity, they demonstrated the heart’s potential early response to radiation. Another study [53] found no changes in perfusion in irradiated myocardium whatsoever, 7 years after radiotherapy, though the study was limited by a lack of baseline control scans. While these imaging studies are in early phases, they contribute towards a better understanding of cardiac toxicity after EBRT and have potential to be used in combination with clinical cardiac function tests to assess toxicity.

### Kidneys

**Fig. 2:**
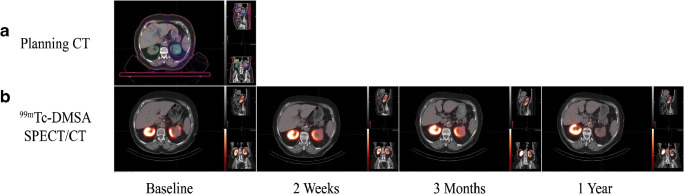
Planning CT (**a**) and longitudinal hybrid ^99m^Tc-DMSA SPECT/CT (**b**) images acquired for a patient who received SBRT for renal cancer. This image was reproduced from Siva et al. with permission [55]

Kidney function and toxicity are generally expressed with GFR. This can be evaluated with radiopharmaceuticals like [^51^Cr]Cr-ethylenediaminetetraacetic acid (EDTA) but is more often characterised by creatinine clearance from blood tests. However, this does not provide information on relative function loss in individual kidneys, or subparts within the kidney. [^99m^Tc]Tc-diethylenetriaminepentaacetic acid (DTPA) dynamic renography may also be used to measure GFR since it is solely filtered by the glomerulus. Scintigraphy or SPECT with [^99m^Tc]Tc-dimercaptosuccinic acid (DMSA) produces static images that can visualise regional kidney function and structure, due to high retention of the tracer in the renal tubules. A study used longitudinal DMSA SPECT/CT (Fig. 2) to evaluate renal dysfunction in patients with renal carcinoma, who received stereotactic body radiation therapy (SBRT) and found that for every 10 Gy of dose delivered, the exponential decline in kidney function was 25–39%, dependant on fractionation scheme [55]. Dynamic renography with [^99m^Tc]Tc-mercaptoacetyltriglycine (MAG3), the most commonly used renal tracer, which is extracted from plasma by the proximal tubules with high efficiency, is used to measure the effective renal plasma flow and can be used as an independent measure of kidney function. Two studies evaluated nephrotoxicity after chemoradiotherapy for gastric cancer using ^99m^Tc-MAG3. One demonstrated a strong correlation between loss of kidney function and volume of the kidney receiving more than 35 and 40 Gy [56]. The other study used the modality to confirm late nephrotoxicity in IMRT was lower than in 3D conformal RT [57]. Most recently, ^99m^Tc-MAG3 renography was used to evaluate function after carbon ion therapy [58].

Kidney toxicity typically occurs in two RNTs: PSMA ligand therapy and DOTA-TATE or octreotide (DOTA-TOC) therapy. In the case of PSMA therapy, this is because the kidneys express the PSMA receptor, whereas in DOTA-TATE therapy, it is because of renal tubular reabsorption of the radioactive peptide. Kidney toxicity can be largely reduced in DOTA-TATE therapy with the infusion of lysine and arginine, which reduces the residence time of the radiopeptide in the renal tubules. As mentioned previously, post-therapy SPECT images can be made to verify biodistribution from ^177^Lu-DOTA-TATE therapy. One study successfully screened a strategy to reduce the infusion time of the aforementioned amino acids by 2 h using this principle [59]. Using these scans to individualise therapy for subsequent cycles by tailoring the dose to the kidneys has also shown to be feasible [60]. Nephrotoxicity in patients who received [^177^Lu]Lu-PSMA, while low, has been reported to be predictable using baseline ^99m^Tc-MAG3 renography. However, this was not found to be the case for ^177^Lu-DOTA-TATE/TOC [61, 62]. Most recently, ^99m^Tc-MAG3 SPECT was used to assess dynamic renal changes from ^177^Lu-DOTA-TATE therapy in mice [38]. Recent years have also seen the development of new renal PET tracers, such as [^68^Ga]Ga-EDTA and [^18^F]fluorosorbitol (FDS), which allow for imaging with better resolution and quantification [63]. Assessment aside, strategies to reduce kidney toxicity in PSMA therapy have also been developed and tested. Mannitol, an osmotic diuretic acting on the proximal tubules, was found to reduce ^68^Ga-PSMA uptake on PET/CT in the kidneys by up to 24% without affecting tumour uptake. However, no significant change in absorbed dose to the kidneys could be calculated in a subsequent ^177^Lu-PSMA therapy study that used planar images for dosimetry [64, 65]. A couple of studies that evaluated the effect of 2-(phosphonomethyl)pentanedioic acid (2-PMPA), a PSMA inhibitor, in mice using ^125^I- and ^111^In-based PSMA ligands, found a near total reduction in PSMA uptake in the kidneys, but tumour uptake was still compromised [66, 67]. Recently, monosodium glutamate (MSG), which competes with the administered radiopharmaceutical for the PSMA receptor, has been tested, and while a 23% reduction in kidney uptake was found, an undesirable 33% reduction in tumour uptake was also seen [68]. The niche of kidney toxicity evaluation with nuclear medicine is a large one with much interest.

### Liver

**Fig. 3:**
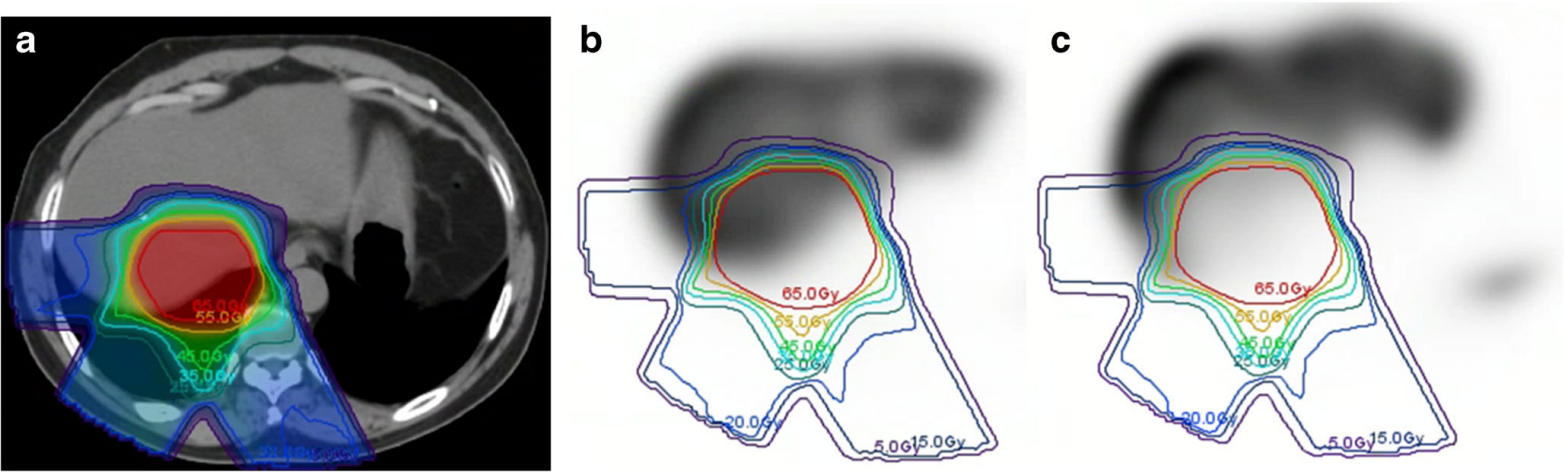
Planning CT (**a**) and longitudinal ^99m^Tc-sulfur colloid SPECT images, baseline (**b**) and 1 month post-treatment (**c**), acquired for a patient who received proton therapy for liver cancer. This image was reproduced from Price et al. with permission [70]

The liver is a frequent site for finding metastases from lung, breast and gastrointestinal cancers. These metastases, as well as hepatocellular carcinoma, can be treated with EBRT (and more specifically SBRT) or selective internal radiation therapy (SIRT or radioembolization). In each of these techniques, liver toxicity (radiation-induced liver disease) is a real risk. An increase in [^18^F]FDG uptake in the liver after chemoradiation (mean SUV_max_ of 5.7 in avid region) has been shown to reflect possible radiation-induced liver disease [69]. Dose response of functional liver from EBRT/SBRT has been assessed with a variety of ^99m^Tc-labelled compounds. A proof of concept study that used [^99m^Tc]Tc-sulfur colloid, particles that are phagocytised by the Kupffer cells in the liver, measured regional dose response of functional liver in patients with hepatocellular carcinoma who received SBRT or proton therapy using SPECT/CT (Fig. 3) [70]. They found high interpatient variability, indicating patient-specific radiosensitivity, which was predictive for long-term toxicity. A study [71] using [^99m^Tc]Tc-iminodiacetic acid (IDA), a hepatobiliary tracer that is extracted from blood by hepatocytes and ultimately excreted into bile ducts, found that baseline and mid-treatment SPECT/CT could predict post-RT regional liver function reserve. Another study [72] used [^99m^Tc]Tc-mebrofenin (an IDA derivative) SPECT in SBRT dose planning to successfully spare liver function, on a single trial patient and achieved excellent target coverage. This has potential to be expanded further. One study showed that SPECT/CT imaging with [^99m^Tc]Tc-galactosyl human serum albumin (GSA), which binds to the asialoglycoprotein receptor present on hepatocytes, could be incorporated into IMRT treatment planning and reduce dose to the functional liver volume [73].

Before SIRT with ^90^Y-labelled resin microspheres, the treatment is often planned with an intraarterial hepatic SPECT/CT using [^99m^Tc]Tc-macroaggregated albumin (MAA), aggregates of human serum albumin around 20–50 μm in size, to assess biodistribution and screen extra-hepatic deposition, which often can occur in the lungs [74]. After administration of the microspheres, a ^90^Y PET scan can also be made to accurately determine dose deposition in the tumour and other regions [75, 76]. This approach illustrates the current relevance of molecular imaging for treatment optimization, to avoid or limit normal tissue toxicity in the liver.

### Lungs

Ventilation-perfusion (V/Q) SPECT is routinely used to assess pulmonary function. V SPECT is widely performed with aerosols like ^99m^Tc-DTPA (0.5–2 μm in size) and Technegas (ultra-fine suspension of ^99m^Tc-labelled graphite nanoparticles), although some centres use ^81m^Kr as well, which is more expensive. Q SPECT is performed with ^99m^Tc-MAA [77–79]. Studies have shown that local changes in Q scans correlate with radiation-induced lung damage from EBRT [80–84], and have noted the value of pre-treatment Q scans in the radiotherapy planning and the prediction of lung injury [85–88], with efforts made to also incorporate V scan data [89]. Recently, with the advent of V/Q PET, using ^68^Ga-tagged radiopharmaceuticals instead of ^99m^Tc, this has been improved upon, given the superior sensitivity and resolution of the technique [90]. One group demonstrated its use in RT planning to reduce dose to functional regions, and also derived a linear dose-effect relationship, finding a 0.7% loss in perfusion and ventilation per Gy [91, 92]. Increased uptake on [^18^F]FDG PET in normal lung tissue after radiotherapy has also been evaluated and attributed to radiation pneumonitis [93, 94]. A linear dose-response relationship was found with some noting the slope varying widely between patients. [95–97]. Some studies also found that pre-radiotherapy [^18^F]FDG uptake values predicted toxicity [27, 98, 99].

### Salivary glands

**Fig. 4:**
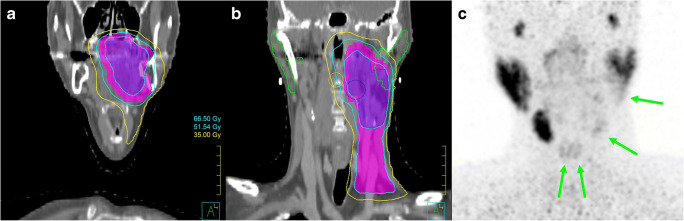
Planning CT (**a**, **b**) and 1 year post-therapy ^68^Ga-PSMA PET image (**c**), acquired for a patient who received RT for tonsillar carcinoma. This image was reproduced from Valstar et al. with permission [106]

Toxicity in salivary glands (parotid and/or submandibular) has been measured using many different radiotracers. [^99m^Tc]pertechnetate scintigraphy has been used for decades to image salivary gland disorders (like Sjögren and Bell’s palsy), and has also been used for evaluating salivary gland function loss after EBRT [15, 100]. The same radiotracer can also be used to make SPECT scans, and studies have used it to better quantify dose-response relationships [101, 102]. Dynamic PET with [^11^C]methionine, which is a surrogate for protein synthesis, has also been used to quantify dose-response relationships on a per voxel basis. The reduction in [^11^C]methionine uptake with increase in dose followed a sigmoid curve and a TD_50_ of 30 Gy was found [103]. Loss of [^18^F]FDG uptake after radiotherapy has also been reported and quantified with dose-response curves, and while it correlated with sialometry and observer-rated outcomes, it failed to do so with patient-reported ones [104, 105]. Recently, salivary gland damage from EBRT dose fields was also visualised with high resolution using ^68^Ga-PSMA PET, as a marker of specific loss of the secretory cells in the salivary glands (Fig. 4) [106]. This shows potential, and it could be used to derive more accurate dose-effect relationships due to the tracer’s high uptake.

**Fig. 5:**
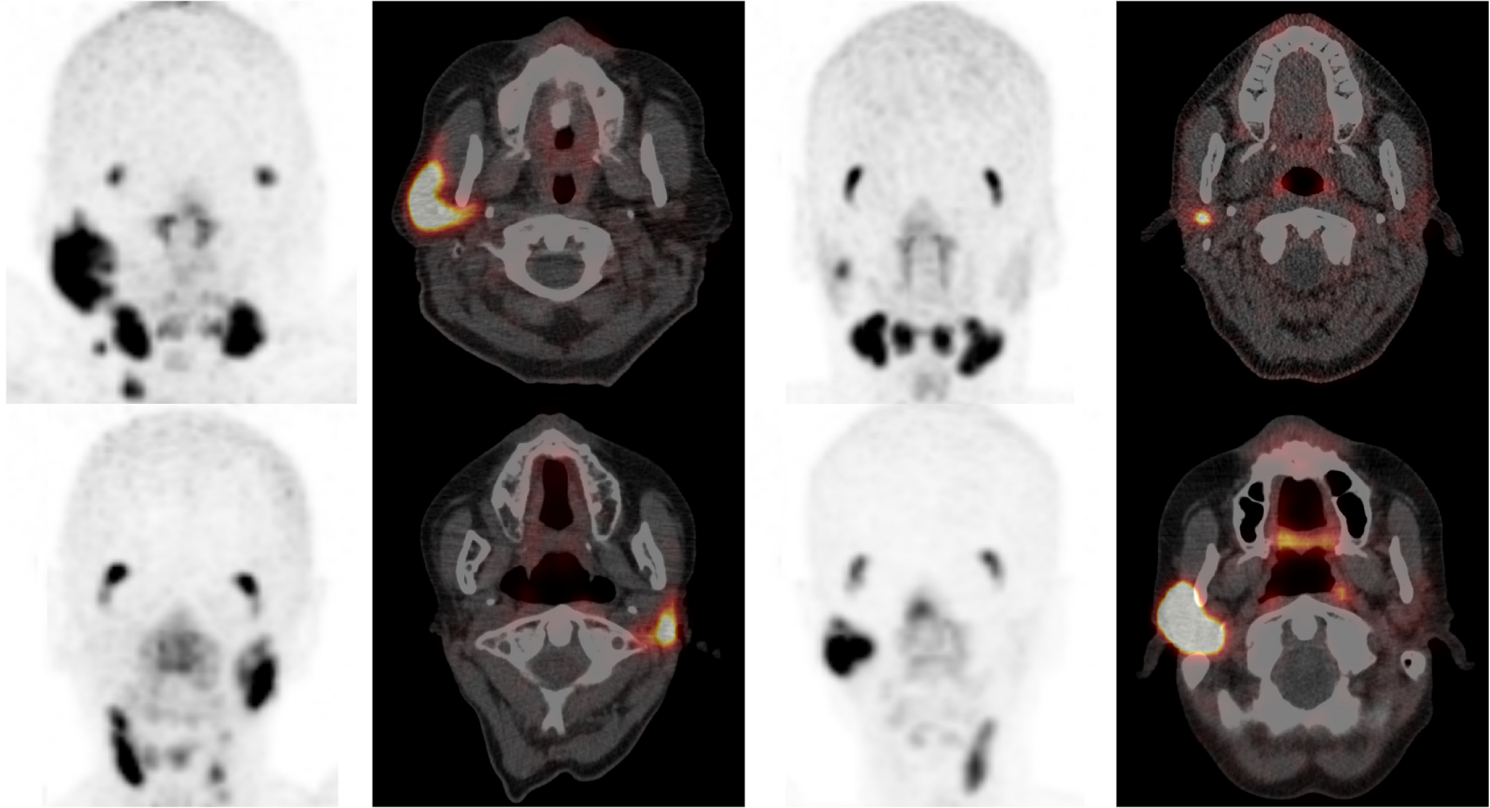
^68^Ga-PSMA PET/CT images acquired for 4 patients with a history of multiple ^131^I therapy cycles. This image was reproduced from Mohan et al. with permission [109]

Salivary gland toxicity occurs in ^131^I therapy, due to the expression of the sodium iodide symporter in the gland cells. Toxicity from ^131^I therapy has been measured in the past using [^99m^Tc]pertechnetate scintigraphy [107, 108] and time-activity curves from these scans, but was also recently demonstrated using ^68^Ga-PSMA PET, exhibiting large intra/interpatient variation (Fig. 5) [109]. Strategies to reduce toxicity such as using sialagogues like lemon juice and pilocarpine [110, 111], and other interventional pharmaceuticals like amifostine [112], have also been tested using the same technique, as well as using ^123^I scintigraphy and ^124^I PET [113, 114]. While most of these strategies were unsuccessful, there is still contention over the effect of stimulation with lemon juice or candy on the uptake of iodine. Toxicity also occurs in the glands in PSMA therapy for metastatic prostate cancer (with ^177^Lu or ^225^Ac), since the salivary glands express the PSMA receptor [22, 29]. Strategies to reduce uptake specifically in the glands have been screened using ^68^Ga-PSMA and [^18^F]DCFPyl (a radiofluorinated PSMA inhibitor) PET/CT. One study superficially cooled the salivary glands with ice packs to reduce perfusion. The reduction in PSMA uptake was, however, insignificant [115]. A study that orally administered MSG [68], having originally found success in a murine model [116], and a study that administered glutamate tablets [65], found a significant reduction in the uptake of PSMA in the salivary glands. Unfortunately, as mentioned before in the case of the kidneys, the co-reduction in uptake in tumours is detrimental. One study injected botulinum toxin into a parotid gland, and found a 64% reduction in PSMA uptake. This is so far the only potentially successful candidate to reduce PSMA uptake in the salivary glands without compromising the tumour uptake. It remains to be seen if this protection strategy is acceptable on a larger scale [117].

### Miscellaneous

The aforementioned organs and tissue types have more than a single tracer associated with their toxicity. There are several other tissues that have been observed to show increased [^18^F]FDG uptake on post-radiation therapy PET scans, due to radiation-induced inflammation. Radiation esophagitis has been shown to correlate strongly with increased [^18^F]FDG uptake on post-therapy PET scans [118–120]. Soft tissues in head and neck, like the glottic and supraglottic larynx, have also shown similar behaviour, and increased uptake correlates with decreased quality of life [121]. Recently, it was demonstrated in a single patient that chest wall toxicity could manifest as increased [^18^F]FDG uptake after intrathoracic SBRT [122].

## Future prospects and conclusion

Toxicity evaluation from radiation treatments has already expanded from mere subjective patient-reported complaints to objective, quantifiable and local measures. Molecular imaging is increasingly providing options for this purpose, with newly developed radiopharmaceuticals or novel applications of already established ones. The emerging trend to move from scintigraphy and SPECT to PET, due to the superior quality and resolution of the modality, further allows for better quantification of dose-effect relationships. Even within PET, there is now a push towards using ^18^F-based diagnostic tracers, rather than ^68^Ga tracers, due to the increase in resolution stemming from decreased positron energy, favourable half-life and cost considerations [123]. [^18^F]FDG is used extensively in tumour imaging, but has shown promise in assessing several toxicities as well. Despite its ubiquity, its lack of specificity, sensitivity and dynamic uptake range makes it nonetheless a suboptimal choice. Its ambivalent response in tissues, exhibiting a reduction in metabolic activity in some while an increase in others from inflammation, can be confounding. For most examples discussed above, superior tracers exist.

With better understanding of dose-effect relationships, individualised EBRT treatment plans with considerations to sparing made based on baseline function, minimising toxicity, show promise. Multiple examples of this are presented above. Such relationships could also be more broadly used to derive dose constraints for EBRT, especially in organs where such limits were derived using questionnaires or lab tests with low reliability. In some of the tissues discussed, SPECT/PET response to radiation damage showed large interpatient variation, essentially measuring the radiosensitivity of the patient. Radiosensitivity in conjugation with dose is a much better predictor of toxicity. If features of baseline scans are capable of capturing this, and mid-treatment response scans if feasible, it would be possible to stratify patients based on risk of developing adverse effects and explore different treatment options.

Since the introduction of radioactive iodine therapy, many more RNTs have emerged. A challenge with RNTs is toxicities due to accumulation in normal tissues. Assessing these toxicities post-therapy with nuclear medicine imaging techniques, using a variety of tracers, has been discussed above. Additionally, pre-therapy scanning with diagnostic analogues to estimate dosimetry, as well as to screen for protective strategies and interventions, is key if newer RNTs are to be introduced clinically. Strategies that work in preclinical models do not always translate suitably. Screening the effect of these interventions relative to a control in patients with diagnostic counterparts before therapeutic doses are attempted has the potential to uncover compromises in tumour dose and unexpected, unintentional biodistribution changes. Moreover, uptake of these tracers in normal tissues, while undesirable in RNT, could be exploited as a measure of function of these unintentional targets. This could then be used to measure function loss of that tissue from other types of treatments, like EBRT, in an objective way.

In conclusion, the application of nuclear medicine for assessing and reducing toxicity is quite well established in some normal tissues, and has seen many new developments in others in recent years. Nuclear medicine imaging can quantify in vivo toxicity and remaining tissue function objectively and in high resolution, to predict and monitor effects of radiation treatments. It can also evaluate strategies that attempt to reduce said toxicities, contributing to minimising the current key drawback of radiation treatments.

## Data Availability

Not applicable.
